# Biological Heterogeneity of Chondrosarcoma: From (Epi) Genetics through Stemness and Deregulated Signaling to Immunophenotype

**DOI:** 10.3390/cancers13061317

**Published:** 2021-03-15

**Authors:** Agnieszka Zając, Sylwia K. Król, Piotr Rutkowski, Anna M. Czarnecka

**Affiliations:** 1 Department of Soft Tissue/Bone Sarcoma and Melanoma, Maria Sklodowska-Curie National Research Institute of Oncology, 02-781 Warsaw, Poland; Agnieszka.Zajac@pib-nio.pl (A.Z.); piotr.rutkowski@pib-nio.pl (P.R.); 2 Department of Molecular and Translational Oncology, Maria Sklodowska-Curie National Research Institute of Oncology, 02-781 Warsaw, Poland; sylwia_krol15@wp.pl; 3 Department of Experimental Pharmacology, Mossakowski Medical Research Centre, Polish Academy of Sciences, 02-176 Warsaw, Poland

**Keywords:** primary bone tumor, cartilage-forming malignancy, chondrosarcoma, mesenchymal stem cell

## Abstract

**Simple Summary:**

Chondrosarcoma (ChS) is the second most frequently diagnosed malignant bone tumor of cartilaginous origin and is generally resistant to standard treatment options. In this paper, we aim to review the current state of the knowledge regarding ChS. We discuss the genetic, epigenetic, and molecular abnormalities underlying its substantial biological and clinical heterogeneity. This review summarizes the critical genetic and molecular drivers of ChS development and progression, contributing to its radio- and chemotherapy resistance. We describe genomic aberrations and point mutations, as well as epigenetic modifications and deregulated signal transduction pathways. We provide an insight into the stem-like characteristics and immunophenotype of ChS. The paper also outlines potential diagnostic and prognostic biomarkers of ChS and recently identified novel targets for future pharmacological interventions in patients.

**Abstract:**

Chondrosarcoma (ChS) is a primary malignant bone tumor. Due to its heterogeneity in clinical outcomes and resistance to chemo- and radiotherapies, there is a need to develop new potential therapies and molecular targets of drugs. Many genes and pathways are involved in in ChS progression. The most frequently mutated genes are isocitrate dehydrogenase ½ (*IDH1*/*2*), collagen type II alpha 1 chain (*COL2A1*), and *TP53*. Besides the point mutations in ChS, chromosomal aberrations, such as 12q13 (*MDM2*) amplification, the loss of 9p21 (*CDKN21*/p16/*INK4A* and *INK4A-p14ARF*), and several gene fusions, commonly occurring in sarcomas, have been found. ChS involves the hypermethylation of histone H3 and the decreased methylation of some transcription factors. In ChS progression, changes in the phosphatidylinositol 3-kinase/protein kinase B/mammalian target of rapamycin (PI3K–AKT–mTOR) and hedgehog pathways are known to play a role in tumor growth and chondrocyte proliferation. Due to recent discoveries regarding the potential of immunotherapy in many cancers, in this review we summarize the current state of knowledge concerning cellular markers of ChS and tumor-associated immune cells. This review compares the latest discoveries in ChS biology from gene alterations to specific cellular markers, including advanced molecular pathways and tumor microenvironment, which can help in discovering new potential checkpoints in inhibitory therapy.

## 1. Introduction

Chondrosarcoma (ChS) is the second most frequently diagnosed primary malignant bone tumor [1], representing a very heterogeneous group of malignant chondrogenic tumors, accounting for approximately 30% of all bone malignancies and characterized by cartilage matrix production [2]. This tumor group is infrequent, and its incidence is 1 per 200,000/year [3]. ChS is characterized by a great variety of histopathology subtypes with specific genetic pathobiology and clinical outcome [4]. The prognosis for most patients with ChS is favorable and correlates with histologic grade and accomplishment of adequate surgical margins upon radical resection [5].

ChS is classified as conventional ChS (the majority, 85–90% of ChS cases), divided into the central and peripheral subtypes; or non-conventional ChS—including periosteal, clear cell, mesenchymal, and dedifferentiated ChS [6]. Myxoid and mesenchymal ChS subtypes are also known as the extraskeletal ChS [2]. Central and peripheral ChS are divided into grades 1, 2, and 3 [7], correlating with tumor malignancy [8]. The 5-year survival of patients with low-grade (grade 1) conventional ChS is 83%; however, in high-grade (grade 3), it does not exceed to 53% [4]. For dedifferentiated ChS, 5-year survival is only 7 to 24% [9]. ChS is highly resistant to chemotherapy and radiation [10], excluding its mesenchymal [11] and dedifferentiated subtypes [12,13]. In conventional, periosteal, and clear cell ChS, surgery is still the only curative treatment method [14]. Most recent studies on the potential use of immune checkpoint inhibitors have not yielded satisfactory results. The response to anti-programmed cell death 1 (PD-1) antibody pembrolizumab treatment was only 20% in dedifferentiated ChS in the SARC028 trial [15].

In the current review, we describe ChS biology, including activated molecular pathways, gene alterations, epigenetics, and immunological features of this disease. We believe that all presented discoveries may help the readers understand the pathology of ChS or design research targeted on finding new potential therapeutic targets and novel drugs to treat this tumor in the future.

## 2. Chondrosarcoma Stem Cell

A significant number of high-throughput analyses and large-scale transcriptomic and proteomic profiling of many types of cancer cells have revealed the presence of a cancer stem cell (CSC) subpopulation within the tumors. CSCs can prominently contribute to an extensive genetic and molecular heterogeneity of cancers and sarcomas (sarcoma stem cells—SSC) [16,17,18]. CSCs/SSCs constitute a relatively rare subset of cells within the tumor; nevertheless, they harbor a high potential for self-renewal and multi-lineage differentiation [19]. They can reinitiate tumor growth and promote metastases formation due to considerably enhanced migration and invasion potential. CSCs/SSCs may invade adjacent and distant healthy tissues [20]. Moreover, increased resistance to standard treatment modalities, such as chemotherapy and ionizing radiation, is one of the most critical features of CSCs/SSCs. Furthermore, CSCs exhibit mesenchymal stem cell (MSC) phenotype [21], and their presence was reported in many sarcoma subtypes, including rhabdomyosarcoma, synovial sarcoma, undifferentiated pleomorphic sarcoma, and osteosarcoma [22,23]. Nowadays, SSCs are considered essential drivers of disease progression and relapse [24] and are therefore important targets for novel oncological therapies [25,26].

**Figure 1:**
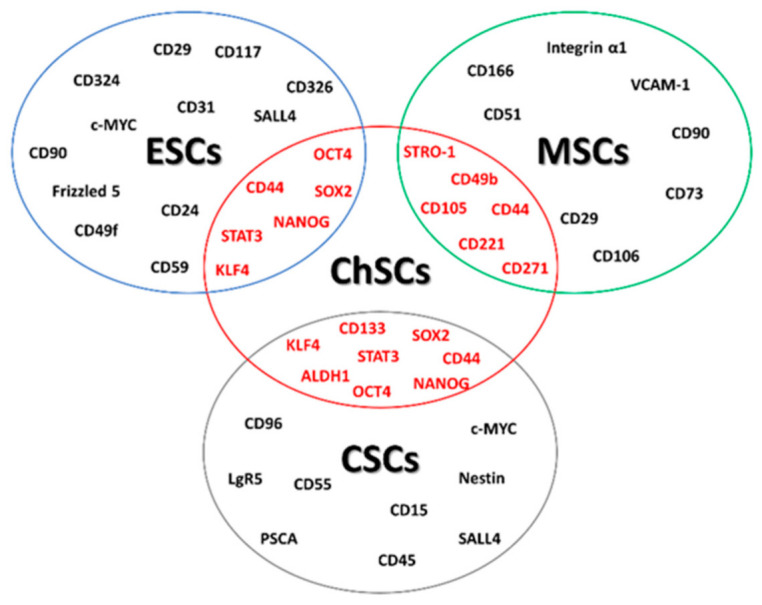
Chondrosarcoma stem cell (ChSC) markers. ChSC markers overlap with embryonic stem cells (ESCs), mesenchymal stem cells (MSCs), and cancer stem cells (CSCs).

Consequently, the treatment targeting SSCs inducing cell differentiation or programmed cell death of this subpopulation is recognized as a promising and encouraging approach [27,28]. A rapidly growing number of data suggest that ChS may originate from transformed MSCs localized in the bone marrow [29,30,31,32]. Stem cells in ChS share some characteristics with MSCs, embryonic stem cells (ESCs), and CSCs (Figure 1).

First of all, stemness or stem-like phenotype of ChS cells is defined by the expression of markers specific for multipotent MSCs, such as CD49b (integrin alpha-2) and CD221 (insulin-like growth factor 1 receptor, IGF1R) [30,33]. Other surface markers of ChS stem cells are CD271, CD44, or CD105. CD271, which is one of the MSC surface markers possibly related to the osteogenic potency of MSCs [34], is upregulated in a highly proliferating subpopulation of ChS cells derived from primary tumor samples [35]. CD271-positive cells, compared with CD271-negative, demonstrated a greater capability for self-renewal, differentiation, drug resistance, and higher tumorigenicity [36]. ChS cell cultures derived from patient biopsies were able to form tumor spheres in vitro and expressed several surface MSCs specific markers, such as CD44, CD105, as well as bone marrow stromal-1 antigen (Stro-1), which is a cell surface marker expressed by stromal elements in human bone marrow-1) [37]. Therefore, ChS cells may be induced to differentiate into at least two mesenchymal lineages—adipogenic and osteogenic [38]. CD44 (phagocytic glycoprotein 1, PGP-1), an MSC protein, is a receptor for osteopontin, hyaluronan, collagens, and matrix metalloproteinases in many different tissues [39]. This protein is overexpressed in progressive ChS, and its expression positively correlates with ChS metastatic potential and poor patient survival [40].

The CD133 antigen, a membrane glycoprotein encoded by the prominin 1 (*PROM1*) gene, is another essential marker characteristic of ESCs and CSCs identified in a subpopulation of ChS cells [41]. CD133-positive cells display stem-like phenotypes and can initiate and support tumor growth in vivo [32]. ChS cell lines derived from clinical samples contained a small fraction of CD133^+^ cells (approximately 5.0–7.8%) with the ability for self-renewal, sphere formation, adipogenic and osteogenic differentiation, and high tumorigenicity in vivo [42]. Significant evidence shows that aldehyde dehydrogenase (ALDH) or CD133 can be considered potentially useful therapeutic targets for eliminating CSCs [43]. Many data suggest the potential prognostic relevance of SSC/CSC marker abnormalities in clinical practice [39,40,44].

ChS stem cells were confirmed to express typical stem-related transcription factors. Upregulated expression of the octamer-binding protein (OCT)-3/4 (also known as OCT4; POU domain, class 5, transcription factor 1, POU5F1) and Nanog Homeobox (NANOG) was found in ChS spheres originating from surgical samples [38]. OCT-3/4 and NANOG regulate the self-renewal, proliferation, survival, and multi-lineage differentiation potential of ESCs. Moreover, these markers not only sustain pluripotency of undifferentiated ESCs but may also act as oncogenes since their overexpression has been identified in several cancers [45,46].

Deregulation of sex-determining region Y-Box (*SOX*) family genes encoding proteins that contribute to cell development, homeostasis, and regeneration [47] was also determined in sarcomas, including ChS. Depletion of SOX2, a pluripotency factor characteristic of ESCs and CSC, resulted in a dramatic decrease in sphere formation and tumor growth initiation. At the same time, its upregulation significantly enhanced pro-tumorigenic activity in vivo. Among sarcomas, the role of SOX2 in tumor initiation and progression has been well characterized in osteosarcoma [44]. Moreover, a correlation of SOX2 expression with tumor grade, invasiveness, and patients’ lower survival was found [44]. SOX9, another well-established pluripotency transcription factor playing an essential role in chondrogenesis, was found to be overexpressed in conventional, mesenchymal, and clear cell ChSs, and downregulation was observed in dedifferentiated ChS [47,48].

Increased ALDH activity is a well-recognized hallmark of hematopoietic stem cells, neural stem cells, progenitor cells, and CSCs in several tumors [49,50]. Highly upregulated ALDH has been found in established human ChS cell lines with enhanced invasiveness, tumorigenicity, and the ability to grow as tumor spheres. Since ALDH is one of the detoxifying enzymes contributing to the oxidation of aldehydes [51], and therefore involved in the scavenging of reactive oxygen species (ROS), ALDH^+^ ChS cells demonstrated a significantly lower ROS level than ALDH^−^ cells, which is characteristic for CSCs [52,53]. Furthermore, ALDH-positive, but not ALDH-negative, cells showed an expression of the Krüppel-like factor 4 (KLF4) protein, which is one of the so-called Yamanaka factors of pluripotency. This may contribute significantly to the maintenance of a stem-like phenotype in ChS cells [54].

Some recent studies have also revealed an essential role of selected signaling pathways and miRNA signaling in ChS SCSs. The phosphatidylinositol 3-kinase/protein kinase B/mammalian target of rapamycin (PI3K–AKT–mTOR) signaling pathway is involved in maintaining stem-like phenotypes. Rapamycin, an mTOR pharmacological inhibitor, significantly increased the level of suppressive miR-34 accompanied by the overexpression of Forkhead box O transcription factor 3 (FOXO3), which resulted in an inhibited proliferation of stem cells [54]. The mTOR inhibition also reduces the number of ALDH^+^ cells and the loss of sphere formation ability in ChS in vitro [54]. Additionally, tumor-suppressive microRNA-34 (miR-34), well-known for the modulation of such target genes as Notch homolog 1 (*NOTCH1*), *C-MYC*, lemur tyrosine kinase 3 (*LMTK3*), and *KLF4* [55], consequently can suppress stem-like characteristics in many types of cancer [56,57,58] and is suggested to influence stemness in ChS. Generally, miR-34 is downregulated in ChS cell lines, and its overexpression significantly reduced the invasive activity of ChS and the spheroid formation ability of ChS cells maintained in vitro [54].

Finally, recent findings from in vitro and in vivo models have reported a significant hypoxic microenvironment involvement via hypoxia-inducible factors (HIFs) in ChS development and progression, including the maintenance of stem-like phenotypes. HIF proteins were found overexpressed in ChS and correlated with the histological grade of the tumor, playing a pivotal role in the promotion of tumor growth, proliferation, metastasis, and the self-renewal potential of stem cells [59,60,61]. Moreover, some members of the HIF protein family can potentially be targeted by a pharmacological approach, and their inhibition was shown to overcome the chemoresistance of ChS and augment standard chemotherapeutics [59].

## 3. Genomic Abnormalities in Chondrosarcoma

Gene aberrations are observed in ChS, and an increased level of gene abnormalities is observed in a higher grade of this sarcoma [6]. For example, the loss of heterozygosity on the 17p1 chromosome region, which results in *TP53* loss, is present in around 25–30% of conventional ChS, mainly in a higher grade [62,63]. Substitution mutations were also found in *TP53* introns and exons 5 to 8 [62,63,64]. Aberrations in genes involved in p53 and retinoblastoma protein (pRb) pathways, such as an amplification of the 12q13 region with mouse double minute 2 homolog (*MDM2*) and the deletion of 9p21 cyclin-dependent kinase inhibitor 2A (*CDKN2A*), were also found in high-grade ChS [6]. The loss of the 9p21 (*CDKN21*/p16/*INK4A* and *INK4A-p14ARF*) region was suggested to play a key role in ChS progression [65].

Gene copy alterations are frequent events in ChS, as is generally the case in sarcomas [66]. In a study by Hallor et al., 59/67 of conventional (both peripheral and central) ChS displayed abnormal DNA copy number in almost one-third of the genome with a median total size of the imbalance of 826 Mb [67]. The most common alterations were found in 2q33.2, 3p21.31, 4q12-q13.1, 5q31.1, 9q13.1, 9q33.3, 12q14.1, and 21q22.11 regions [67]. Other altered genomic regions contained matrix metalloproteinase (*MMP*) and exostosin glycosyltransferase (*EXT*) genes. In the same study, in more than 25% of cases, copy number alterations included a gain of chromosomes 5, 12, and 19 to 22 and the loss of parts of chromosomes 1, 4, 6, 9 to 11, 14, and 17 and the whole 13q chromosome arm [67]. For ChS large variety of cytogenetic abnormalities are known. Nearly 200 karyotypes were reported in ChSs [68].

In the cytogenetics of sarcomas, gene fusions are significant and are essential markers in diagnostics [69]. Among ChSs, the best-known translocations, based on many studies [70,71,72,73,74], are defined for extraskeletal myxoid ChS (excluded from this review), while only a few translocations have been discovered in mesenchymal ChS [75,76] (Table 1).

## 4. Mutations in Chondrosarcoma

In ChS, several repetitive point mutations have been described. The most common are mutations in *IDH1* and *IDH2* genes, which encode isocitrate dehydrogenase 1 and 2, an enzyme that converts isocitrate to α-ketoglutarate (αKG) [77,78]. Somatic mutations of *IDH1* and *IDH2* result in higher production of 2-hydroxyglutarate (2HG), one of the oncometabolites [79,80] from αKG. Moreover, the *IDH1* mutation affects the intracellular cholesterol synthesis pathway, which can be a potential therapeutic target in ChS [81]. The study by Amary et al. in 2011 indicated the hot spot mutations in arginine (R132C, R132H in *IDH1,* and R172S in *IDH2*) in cartilaginous tumors, including ChS [82]. Mutated *IDH1/2* genes were observed in 38% of peripheral ChS [78] and in 59% of central ChS—a subtype in which this mutation is mainly observed [82,83]. In high-grade conventional ChS, the R140-*IDH2* gene mutation has also been reported [78]. In all grades of conventional central ChS and in the dedifferentiated ChS, missense mutations of *IDH1*/*2* genes have been reported [83]. On the contrary, the *IDH2* mutations were more often observed in high-grade ChS and dedifferentiated ChS, but not in low-grade ChS [78,84]. What is more, *IDH* mutations were correlated with DNA methylation and ChS progression [84].

The second typical mutations in ChS are reported in the *COL2A1* gene encoding the alpha-1 chain of type II collagen fibers in cartilage. These mutations were identified in 37% of ChS [10,83,84]. The studies indicated truncating essential splice-site and missense mutations in high-grade conventional ChS and in dedifferentiated ChS. These mutations cause fundamental perturbation of extracellular matrix (ECM) deposition and signaling that can contribute to oncogenesis by abrogating normal differentiation programs in cartilage tissue [83].

Other common mutations in ChS were reported in genes responsible for the cell cycle regulation [85]. Mutations in the *TP53* suppressor gene occur in 20–50% cases of ChS, both in conventional, dedifferentiated central ChS and peripheral ChS [10,83]. *TP53* is one of the most frequently mutated genes in human cancers [86,87]. More specific in ChS, *TP53* mutations were found in about 30–50% of conventional ChS in all three grades [62,78,88], but their presence was also reported in clear cell ChS [89]. The *TP53* alterations, including insertions, deletions, and single nucleotide variants (SNV), resulted in silent, nonsense, and splice-site mutations or frameshifts [62]. What is more, a strong correlation between the overexpression/alteration of *TP53* and metastasis or histologic grade of the tumor was found [62]. The presence of *TP53* mutations in almost all grade III ChSs suggests their role in ChS progression [6,90]. The p53 loss of function may play a role in the transformation to a highly malignant dedifferentiated ChS subtype [70]. Other genes involved in cell cycle regulation often mutated or loss in ChS are *CDKN2A* and cyclin-dependent kinase inhibitor 2B (*CDKN2B*) [70,85], and *RB1* (retinoblastoma 1) [83].

Moreover, the protein patched homolog 1 (*PTCH1*) nonsense and missense mutations were reported in central conventional and dedifferentiated ChS [83,85]. This gene is involved in the activation of the hedgehog pathway, an early event in the tumor formation and progression process [90]. Moreover, deletions in phosphatase and tensin homolog (*PTEN*) and tuberous sclerosis 1 (*TSC1*) genes in central ChS are observed [83,85]. Besides the somatic mutations in peripheral secondary ChS, germline truncating mutations in exostosin glycosyltransferase 1 and 2 (*EXT1*/*EXT2*) genes, involved in chondrocyte differentiation [6], were also observed [83,90]. The *EXT* gene mutations were mostly inactivating (nonsense, frameshift, or splice-site mutations), which can cause premature termination and the loss of protein functions [83,85].

Although several genes are frequently mutated in ChS, the tumor mutational burden (TMB), defined by the number of somatic mutations per megabase (Mb), is at a low level (<5 mutations/Mb) [85]. On the other hand, the study of a single *COL2A1* gene showed a TMB range from 1 to 115 per case [83]. Among all indicated somatic mutations in this gene, several mutations—missense, nonsense, splice-site, and synonymous mutations, indels, and substitutions in microRNAs—have been identified [83]. In this case, the number of somatic mutations was more than two times higher in dedifferentiated and grades 2 and 3 ChS, compared to grade 1 ChS. According to this, there is still a need to conduct more detailed research in this area in the future [83].

## 5. Epigenetics of Chondrosarcoma

Recent rapidly growing evidence suggests that epigenetic mechanisms and their deregulation contribute to the development and/or progression of ChS. A well-known mechanism underlying DNA and histone methylation alterations is an accumulation of 2HG (2-hydroxyglutarate) induced by mutations in *IDH1*/*2* in cancer cells, as mentioned above [91]. For example, the *IDH2* mutation has been shown to induce the 2HG-dependent DNA hypermethylation in ChS cells, inhibiting mesenchymal differentiation [66]. Treatment with a 5-azacytidine can potentially reverse this blockage of the differentiation. A high level of the 2HG also leads to the competitive inhibition of the Ten-Eleven Translocation (TET) family of DNA demethylases and the Jumonji-C domain-containing family of histone lysine demethylases (JHKDMs).

Nevertheless, since only approximately 50% of all ChSs bear the *IDH* mutations [82], some data suggest that the level of 5-methylcytosine (5-mC) and 5-hydroxymethylcytosine (5-hmC) is highly variable in ChS, possibly not related to the mutational status of *IDH*, and no longer directly dependent on the inhibition of TETs by 2HG. Hypermethylated histone H3 (especially trimethylation of histone H3 at lysine 4 (H3K4me3), trimethylation of histone H3 at lysine 9 (H3K9me3), and trimethylation of histone H3 at lysine 27 (H3K27me3) has been observed irrespectively of the *IDH* mutation status in ChS [92]. Ongoing clinical trials are evaluating the clinical activity of novel IDH inhibitors. Enasidenib (AG-221), an oral IDH2 inhibitor, is currently being tested in phase I/II studies in patients with ChS with the *IDH2* mutation (NCT02273739). Inhibitors of IDH— vorasidenib (AG-881) and ivosidenib (AG-120)—are also being evaluated in phase I studies in ChS with the *IDH1* and/or *IDH2* mutation (NCT02481154/NCT02073994). A combination of metformin with chloroquine in ChS patients with *IDH1*/*2* mutations is also undergoing clinical trials (NCT02496741). Additionally, the DNA methylation pattern and expression of H3K4me3, H3K9me3, and H3K27me3 marks were not altered after the long-term treatment of ChS cells in the in vitro with mutant IDH1 inhibitor AGI-5198 (N-[2-(cyclohexylamino)-1-(2-methylphenyl)-2-oxoethyl]-N-(3-fluorophenyl)-2-methyl-1H-imidazole-1-acetamide) [93].

An alternative mechanism(s) of epigenetic regulation may be involved in ChS development and progression. Interestingly, some data suggest that DNA methylation patterns may be modulated by the microenvironment. Differences in CpG-methylation status of the *Satellite 1* promoter were demonstrated between swarm rat model of human ChS (SRC) cells from different transplantation locations and normal rat cartilage [94]. Several reports have shown aberrant patterns of DNA methylation: hyper- and hypomethylation (global methylation and individual genes). They were followed by silenced or elevated expression of several genes involved in the development of ChS through many signaling pathways, such as cell cycle progression, cell-cell interactions, cell adhesion, and apoptosis. Some tumor-related genes were identified as hypermethylated in human ChS cells.

### 5.1. Hypermethylation of DNA

Tumor suppressor gene *p16INK4a,* which encodes CDKN2A, an inhibitor of cyclin-dependent kinase (CDK) 4/6 engaged in the regulation of phase G1 progression and contributing to growth arrest in deregulated cells [48], was found to be hypermethylated within CpG islands in the promoter in ChS [95,96]. Moreover, *p16INK4a* and E-cadherin (*CDH1*)—a gene associated with cell adhesion—are often hypermethylated in both low-grade chondroid and highly malignant anaplastic components of dedifferentiated ChS [96]. However, hypermethylation in the promoter region of fragile histidine triad (*FHIT*), one of the most frequently changed genes in human tumors related to apoptosis and cell cycle regulation, was found only in a highly malignant dedifferentiated ChS compartment [96]. The abnormally hypermethylated promoter of runt-related transcription factor 3 (*RUNX3*) and its decreased expression was shown, leading to significantly increased proliferation and inhibited apoptosis of ChS cells in vitro. *RUNX3* is a well-known tumor suppressor responsible for the modulation of cell cycle arrest and apoptosis cell death. Methylation of its promoter may also affect clinical outcome since the loss of *RUNX3* expression correlates with poor prognosis in patients with ChS [97]. The aberrant DNA methylation pattern was also identified in the promoter region of *3-OST*, encoding 3-O-sulfotransferase 2 (HS3ST2) involved in the biosynthesis of heparan sulfate (HS). The pharmacological inhibition of DNA methyltransferase (DNMT) in the HEMC human ChS cell line in vitro with hypomethylating agent 5-aza-2′-deoxycytidine (decitabine) restored expression of a hypermethylated *3-OST* gene, which resulted in decreased cell proliferation, invasiveness, and improved cell adhesion [98]. The higher methylation level of the nicotinate phosphoribosyltransferase (*NAPRT1*) promoter, coding for a nicotinic acid phosphoribosyltransferase (contributing to the synthesis of NAD^+^), was found in high-grade ChSs.

Moreover, ChS cells are also sensitive to pharmacological inhibition of nicotinamide phosphoribosyltransferase (NAMPT) since a strong decrease in viability and invasion was observed in vitro. Potentially, patients with a high-grade of ChS and silenced expression of *NAPRT1* may benefit from treatment with inhibitors of NAD^+^ synthesis [99]. Epigenetic silencing by hypermethylation of secreted frizzled-related proteins family (*SFRP5*) promoter was found in ChS cell lines, leading to a considerable decrease in its expression. SFRP proteins are well-known competitive inhibitors of the Wnt signaling cascade [100]. Treatment with the DNA-demethylating agent decitabine prominently inhibited ChS cell proliferation by cell cycle arrest in vitro and inhibited the growth of xenografts and lung metastasis in vivo. Following the reduced methylation of the *SFRP5* promoter, the inactivation of the Wnt/β-catenin pathway, the reduced expression of mesenchymal markers N-cadherin and vimentin, and the increased expression of epithelial marker E-cadherin associated with the induction of mesenchymal-epithelial transition (MET) were observed in ChS [101].

### 5.2. Hypomethylation of DNA

Hypermethylation is one of the mechanisms of inactivating gene expression, contrary to hypomethylation. The global loss of DNA methylation, including hypomethylation in repetitive DNA sequences such as *Satellite 1* and *LINE-1*, was induced by 5-aza-2′-deoxycytidine in SRC cells. The decreased methylation level in promoter regions of Midkine growth factor (*MDK*) and pluripotent transcription factor *SOX2* was also observed. The loss of methylation within CpG sequences after the inhibition of DNMT contributed to increased invasive and migratory activity in vitro and the progression of cancer in vivo [102]. The loss of CpG methylation was associated with the increased expression of two epithelial-specific markers, *Maspin,* and *14-3-3σ,* during the development and progression of ChS. Conversely, no expression of *Maspin* and *14-3-3σ* was found in normal chondrocytes following the hypermethylation of gene promoters. In contrast, the treatment of chondrocyte cells with decitabine led to an activated expression of both epithelial-specific markers. These results demonstrated epigenetic mechanisms regulating and triggering the mesenchymal to epithelial transition (MET) in ChS [103]. *P73*, belonging to the *TP53* gene family and considered a tumor suppressor [104], is also highly methylated within the promoter sequence, resulting in silenced expression of p73 in ChS cells. The inhibition of DNMT by decitabine in vitro led to a significant loss of DNA methylation and consequently to the upregulation of *p73,* which may modulate the expression of some genes targeted by p53. Moreover, a correlation between methylation of the *p73* promoter and the grade of ChS was found. A prominently lower methylation level was observed in less malignant grade 1 tumors compared with those of stages 2 and 3 [105]. However, there is still a very limited number of reports on the hypomethylation status of individual genes in ChS, supporting a need for further study.

Many human cancers exhibit epigenetic aberrations in DNA and in histone modifications and expression of histone deacetylases (HDACs) [106,107,108]. Therefore, HDAC inhibitors have attracted attention as a new class of potential anticancer agents [109,110]. One of the epigenetic regulators with potential significance in ChS may be sirtuin 1 (SIRT1), a member of the sirtuin protein family belonging to the class of HDACs, which showed significant deregulation in ChS. The aberrant expression of SIRT1 regulated tumor cell metastatic capacity through the promotion of epithelial-mesenchymal transition (EMT). Additionally, a correlation between SIRT1 expression, tumor progression, and prognosis has also been found in patients with ChS [111]. The effects of some pharmacological HDAC inhibitors in ChS were studied in vitro and in vivo. Small molecule agents such as sodium valproate (VPA) and trichostatin A (TSA) inhibited the proliferation of ChS cell lines, induced cell cycle arrest, and suppressed tumor growth in vivo [112]. Similarly, suberoylanilide hydroxamic acid (SAHA) treatment resulted in the promotion of apoptosis and the autophagy of ChS cells and tumor growth arrest in animal models of ChS [113], whereas depsipeptide also induced the differentiation of ChS [114].

The DNA methylation status of specific genes may be presumed as a prognostic biomarker in ChS. Furthermore, sarcoma-related epigenetic modifications of DNA and histones are potentially reversible. Therefore, they may be targeted by pharmacological inhibitors, consequently opening up novel opportunities for a promising and powerful therapeutic approach in the treatment of ChS. However, some conflicting data from in vitro and in vivo models show not only suppression but also the progression of sarcoma upon such intervention. At this point in time, there still is an unmet need for more research in the field before the potential implementation of DNMT inhibitors into the therapy of patients with ChS.

## 6. Deregulated Signaling Pathways in Chondrosarcoma

The development, and progression of ChS, are accompanied by an accumulation of genetic and epigenetic alterations in the cells. These changes result in prominent dysregulations and aberrations in many signaling pathways, including cell cycle regulators, oncogenes, and tumor suppressors.

### 6.1. Tumor Suppressor pRb and p53 Pathways

Large-scale transcriptomic and proteomic analyses, including whole-exome sequencing and immunohistochemistry, have revealed common aberrations in tumor suppressors and cell cycle regulators in ChS cells [10,115,116]. In this process, many genes, such as *CDK4* and *MDM2,* players in the pRb and p53 pathways, are involved [116]. Degradation of p53 protein through MDM2 was observed to be associated with tumor progression in a subset of central ChSs [116]. Other research has proved that p53 deficiency can cause ChSs to arise from benign lesions, such as enchondroma [117]. Since the expression of *MDM2* was found to be upregulated in one-third of high-grade ChS and correlated with increasing histopathological grade [116], the inhibition of interactions between p53 and MDM2 by small-molecule agents (e.g., RG7112) restoring the function of p53 may be a rational therapeutic intervention in ChS [118].

The second of the most important signaling pathways perturbed in the majority of cases of conventional and dedifferentiated high-grade ChS is the pRb pathway [116]. pRb inhibits the progression of cells with damaged DNA to the S phase of the cell cycle via binding to the E2F transcription factor [119,120]. Dysregulations of the pRb pathway may include a loss of the heterozygosity (LOH) of the *RB1* gene in grade 3 and dedifferentiated ChS [115], the decreased expression of *CDKN2A*/p16, or the augmented expression of CDK4 or cyclin D1 [116]. Therefore, pharmacological blocking of CDK4/6 is suggested as a reasonable approach to potentially be applied in the treatment of advanced ChS since several CDK4/6 inhibitors (palbociclib, ribociclib, and abemaciclib) have recently been approved for the therapy of metastatic breast cancer [121].

### 6.2. PI3K–AKT Signal Transduction

The data from kinome profiling of several human sarcomas have shown the PI3K–AKT signaling pathway is strongly activated in many cancers via frequent mutations in specific proteins of this pathway [122]. Activation of serine-threonine kinase AKT is triggered partly by the binding of phosphatidylinositol (3,4,5)-trisphosphate (PIP3), which is generated by a lipid kinase PI3K. The PI3K–AKT is a well-established regulator of multiple biological processes, such as cell survival, proliferation, growth, metabolism, and programmed cell death. AKT is the most activated kinase in ChS cells [123,124]; however, specific activating mutations within this pathway are infrequent [125]. Numerous novel therapeutic agents blocking PI3K–AKT signaling are currently undergoing clinical trials for the treatment of several human solid tumors (pan-class I and isoform-specific PI3K inhibitors, AKT inhibitors) [126]. They may potentially be used in the treatment of ChS.

### 6.3. The SRC Signaling Pathway

Another aberrantly activated kinase pathway identified during kinome profiling of human ChS cells was the SRC (Proto-oncogene tyrosine-protein kinase Src) signaling cascade, well-known for playing an essential function in embryonic development and cell growth [123,127]. The SRC pathway is an upstream regulator of PI3K–AKT signaling since its activation not only promotes sarcoma cell survival, proliferation, and migratory potential but may also increase the level of phosphorylated AKT, contributing, therefore to the activation of pro-survival pathways [33,127]. The SRC signaling cascade can be targeted by dasatinib (BMS-354825), a small molecule tyrosine kinase inhibitor that may also block Abelson tyrosine-protein (ABL) kinases [127]. Dasatinib is effective in the treatment of some solid tumors, chronic myelogenous leukemia, and Philadelphia chromosome-positive acute lymphoblastic leukemia [123,127]. Inhibition of the SRC pathway by BMS-354825, at nanomolar concentrations, significantly reduced the cell viability and survival of ChS in vitro, although no induction of caspase 3-mediated apoptosis was found [123]. Moreover, sensitization of ChS cell lines with mutant p53 to doxorubicin by dasatinib treatment was observed, with prominent inhibition of migration and apoptosis induction in vitro. These data clearly demonstrate that the SRC signaling cascade is essential for the chemoresistance of ChS cells. Since approximately one-third of ChSs harbor mutations in *TP53*, especially high-grade tumors, inhibition of the SRC pathway may potentially be useful in overcoming resistance to standard chemotherapeutics [127]. However, the results of the phase II trial of single-agent dasatinib in previously treated patients with high-grade sarcomas revealed no clinical benefits [128].

### 6.4. The mTOR-S6K Pathway

Enhanced signaling of the mTOR has been observed in many human sarcomas. mTOR is a serine/threonine kinase and a downstream of the PI3K–AKT signaling cascade involved in the modulation of cell cycle progression, cell growth and proliferation, autophagy, angiogenesis, and several other metabolic processes by the integration of intracellular nutrient status and extracellular signaling [129,130]. Rapamycin (sirolimus (SIR)) is a macrocyclic triene antibiotic and a well-established inhibitor of mTOR signaling. Sirolimus inhibits phosphorylation and thus activates S6 kinase, which is the downstream effector of mTOR. Decreasing the level of phosphorylated pRb may lead to cell growth arrest in the G_1_/S phase. The significantly increased phosphorylation of S6 protein has been observed in 44–69% of dedifferentiated and conventional ChS clinical specimens, and this effect was dependent on the histological grade of the tumor [131]. Some preclinical models have shown an important role of the mTOR pathway in the progression and invasion of ChS. Proline-rich polypeptide 1 (PRP-1) cytokine in high-grade ChS cells reduced mTOR activity and downstream protein c-Myc, followed by a significant anti-proliferative effect [132]. Treatment of ChS cells with rapamycin decreased cell migration and expression of MMP-13, leading to reduced metastatic potential [133]. Simultaneously, irregularities of c-MYC appear to occur in the early stages of tumorigenesis in all ChSs. The overexpression of metalloproteinases MMP-2, MMP-MT1, and tissue inhibitor of metalloproteinases 2 (TIMP2) and the abnormal methylation of p16 and E-cadherin are present in anaplastic cells of dedifferentiated ChS [6]. Pharmacological blocking of the mTOR pathway with dactolisib (BEZ235, NVP-BEZ235), a dual pan-class I PI3K-mTOR inhibitor, dramatically inhibited the growth of ChS cell lines and induced cell cycle arrest at the G_1_ phase, but no apoptosis was observed in vitro. Additionally, dactolisib affected the growth of ChS cells more potently than rapamycin. Moreover, considerable tumor inhibition was also observed in a murine xenograft model after treatment with BEZ235 [131]. In a rat model of ChS, another mTOR inhibitor, everolimus, was demonstrated to decrease cell proliferation. However, apoptotic cell death was not induced. Everolimus suppresses tumor progression and delayed local post-resection recurrence of the disease [134]. Several reports have also shown the clinical benefits of treatment with mTOR inhibitors in patients with ChS. A combination of SIR with cyclophosphamide (CTX) in metastatic, inoperable myxoid ChS resulted in an extraordinary response to the therapy [135]. Similarly, a combined treatment of SIR and CTX at low doses in patients with unresectable conventional ChS led to stabilization of the disease for at least six months in most patients (60%) and seemed to have significant clinical relevance [136].

### 6.5. Hedgehog Signaling Pathway

The Indian Hedgehog Homolog (IHH) pathway and parathyroid hormone-related peptide (PTHrP) pathway play a vital role in the differentiation of healthy chondrocytes, and it has been proven that constitutive IHH signaling plays a key role in the pathogenesis of ChSs. IHH regulates the proliferation and differentiation of chondrocytes through the direct regulation of glioma-associated oncogene (GLI) transcription factors and by regulating PTHrP ligand expression [117]. Abnormal activation of this pathway leads to continuous signals from IHH that induce chondrocyte proliferation and the secretion of PTHrP from chondrocytes into the extracellular matrix [137]. By auto- and paracrine signaling, PTHrP mediates the inhibition of chondrocyte differentiation and apoptosis, thus maintaining cells in the state of cell division [138]. While preclinical data on the activity of IPI-926 (saridegib—an oral hedgehog pathway inhibitor) indicated this compound’s good activity, clinical data from a phase II study in patients with advanced ChS were not satisfactory [83,139]. Similarly, vismodegib (GDC-0449) treatment assessed in a phase II study did not bring the expected results. The median progression-free survival (mPFS) was only 3.5 months, and the median overall survival (mOM) was 12.4 months [140]. These disappointing clinical outcomes may indicate a ligand-independent activation of the Hh pathway in ChS, which may occur in the case of a loss of PTCH function mutation or smoothened, frizzled class receptor (SMO) mutation, causing a loss of function and activation of the downstream pathway [138].

The aberrant activation of several signaling cascades in ChS may suggest their important tumor pathogenesis and progression function. Therefore, frequently deregulated signaling pathways harbor potential targets for treating patients with high-grade, metastatic, and inoperable ChS. The novel therapeutic approach may be opened up by targeting aberrantly expressed pathways with small molecule pharmacological inhibitors and/or in combination therapy.

## 7. Chondrosarcoma Microenvironment

In a ChS microenvironment, similarly to other tumors, tumor-infiltrating lymphocytes (TILs) and tumor-associated macrophages (TAMs) play a pivotal role [141]. TILs infiltration is associated with inflammatory and anti-tumor responses, in contrast to TAMs [142]. Many studies have shown the correlation between immune infiltrate composition, tumor aggressiveness, and patient survival in conventional ChS [141,143]. In a study by Richert et al., CD8+ lymphocytes and CD163+ macrophages were observed in conventional and dedifferentiated ChS, mainly in the peripheral area of the tumor tissue, and their peripheral location was associated with disease progression [143]. However, the infiltration of CD3+ and CD8+ T cells or CD68+ and CD163+ macrophages was not correlated with patients’ clinical features, such as age, gender, and tumor localization [143]. The highest density of CD163+ tumor-associated macrophages type 2 (M2) was observed in the high-grade ChS [143]. These results suggest the M2 cells participate in ChS progression through their role in angiogenesis, migration, and invasion [143,144]. M2 presence is associated with the increased expression of indoleamine 2,3-dioxygenase 1 (IDO). IDO is produced by activated macrophages and is involved in the inhibition of the immune response [145]. Besides CD163+ cells, the researchers also observed a population of CD68+, signal-regulatory protein alpha (SIRPA), and colony-stimulating factor 1 receptor (CSF1R+) TAMs, which are related to the metastatic status at diagnosis and the poor survival of patients with dedifferentiated ChS. [143].

On the contrary, the presence of CD3+, CD4+, and CD8+ TILs is correlated with less invasive ChS phenotype and longer overall survival of the patients [146]. Although the suppressive role of CD8+ cells in tumor development is quite clear, CD4+ cells can interact dually and convert from anti-tumor to pro-tumor role [147]. As observed in dedifferentiated ChS, another population of immune cells are regulatory lymphocytes, such as CD3+ Forkhead box P3 (Foxp3)+ cells. These cells were more often observed in metastatic tumors in comparison to the primary ChS tumors [148,149]. Helper T cells (CD3+CD8−), in contrast to regulatory T cells, are more often observed in primary tumors but not in metastases [148]. The same study demonstrated the presence of markers of activated and immunosuppressive macrophages—CD163+CD14+—both in the primary and metastatic tumors. [150]. Furthermore, Richert et al. showed that, in both conventional and dedifferentiated ChSs, TAMs are found at a higher density than TILs [143].

**Figure 2:**
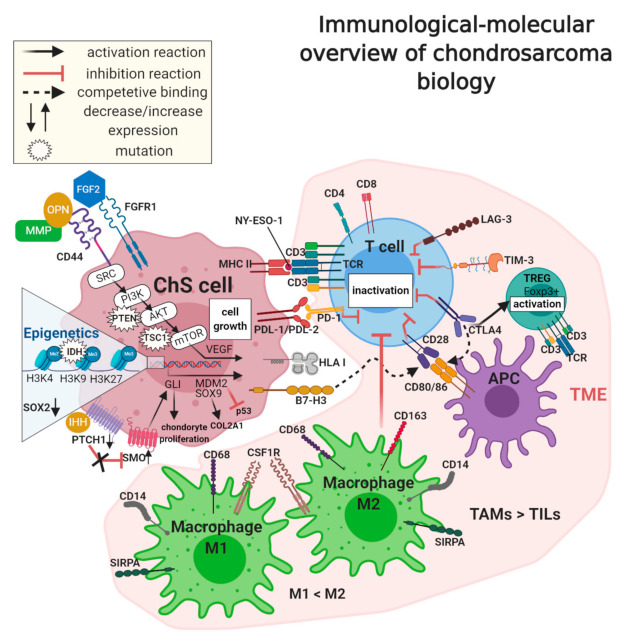
Immunological-molecular overview of chondrosarcoma (ChS) biology. Immune cell markers (PDL-1/2, B7-H3, and MHC), mesenchymal stem cell markers (CD44), and the tumor microenvironment (TME), in which the primary role is played by tumor-infiltrating lymphocytes (TILs) and tumor-associated macrophages (TAMs), especially the M2 type, participate in ChS pathogenesis and immunosuppression. Other important factors involved in ChS progression are deregulations in molecular pathways (PI3K/AKT/mTOR and hedgehog), epigenetic modifications (trimethylation of H3K4, H3K9, H3K27), and gene mutations (*PTEN*, *TSC1*, *IDH*), which together characterize the tumor.

Although ChS immunophenotype data is not extensive, several markers involved in the immune escape of ChSa were described (Figure 2). PD-1 and PDL-1/PDL-2 (programmed cell death ligand-1, B7-H1/programmed cell death ligand-2, B7-DC) are expressed in tumor cells or tumor-associated lymphocytes and macrophages [145,148,150]. Both PD-1 and PDL-1/PDL-2 are expressed in ChS [151,152]. The PDL-1 expression has been correlated with poor survival [143,150,151]. PDL-1 binds to PD-1 on TILs and inhibits T-cell functions, resulting in tumor immune escape [142]. The PD-1/PDL-1 interaction also decreases cytokine production and induces anergy and apoptosis of T cells [142,150]. PD-1 is also expressed on B cells or NK cells and play a role in their decreased activity during tumor growth [145]. The increased expression of PDL-1 was reported in dedifferentiated ChS (>40%), and it was associated with human leukocyte antigen 1 (HLA-1) expression [143,148]. The PD-1+ T cells were observed in both conventional and dedifferentiated ChS at a similar level; however, the PD-1 expression correlated with PDL-1+ tumor cells only in the case of the dedifferentiated subtype [143]. On the other hand, in other studies, PDL-1 was observed in 1% of conventional ChS [153]. PDL-1 expression was also significantly associated with poor overall survival in ChS and osteosarcoma [151], and it was significantly related to a younger age (<30 years), a larger tumor size (>10 cm), an advanced tumor grade, and an earlier recurrence. In comparison, PDL-2 was linked only with an earlier recurrence and a lower age of patients [152]. The association of these ligands with a higher clinical stage, the presence of metastases, a higher histological grade, poor differentiation, and tumor necrosis has also been reported in many soft tissue sarcomas [154].

In the downregulation of immune response, some exhaustion T cell markers are also important, such as T cell immunoglobulin and mucin domain-containing protein 3 (TIM-3), as well as CD223, lymphocyte activation gene-3 (LAG-3). TIM-3 is detected on many types of immune cells, including T and B cells, regulatory T cells, dendritic cells, macrophages, natural killer cells, and mast cells [155]. Besides the increased expression of TIM-3 on tumor-associated lymphocytes [155,156], the expression of TIM-3 was also observed on cancer and sarcoma cells [157], including conventional and dedifferentiated ChS [143]. TIM-3 co-expression with PD-1 can also inhibit T helper 1 cells [145]. Furthermore, TIM-3 expression in NK cells was correlated with disease progression in many cancers [155]. A similar observation has been made with respect to LAG-3, expressed by tumor-associated lymphocytes, which also correlated with PDL-1+ expression [158], including co-expression in ChSs [143]. Blockade of LAG-3 and/or TIM-3 proteins can be a novel potential therapy in ChS. This has been already tested in the first clinical trials, including these with a combination of TIM-3, LAG-3, and PD-1 inhibitors [155,158].

The PD-1/PDL-1 interaction inhibits T cell activation directly, while the inhibitory effect of the next important immune checkpoint, cytotoxic T-lymphocyte-associated protein 4 (CTLA-4), has another mechanism of action [150,159]. This receptor is expressed on previously activated T cells, for example, in regulatory lymphocytes or CD8+ effector T cells [150]. The inhibition of the immune response, in this case, is caused by competition between CD28 and CTLA-4 binding to B7-1/2 (CD80/86) [150]. After T cell activation by CD28 and CD80/86 interaction, CTLA-4 is transported to the T cell surface, where it binds to B7-1 and B7-2 with greater affinity than CD28. As a result, it downregulates the immune response [150]. CTLA-4 plays the primary role in decreasing immune response by inhibiting helper T cell activation and enhancing regulatory T cell (TREG) immunosuppressive activity [145,150]. The CTLA-4 receptor blockade increases CD8+ T cell activation and depletes regulatory T cell action, which has been successfully used in the treatment of melanoma and carcinomas [160]. In conventional and dedifferentiated subtypes of ChS, CTLA-4 was observed at a low level [143].

Another receptor, connected with CTLA-4 and overexpressed in many tumors, is B7 homolog 3 (B7-H3, CD276). It also competitively binds CD28 and consequently inhibits activation, proliferation, and cytokine production of T cells [150]. B7-H3 overexpression was observed in conventional and dedifferentiated ChS at the same level [143]. Moreover, high expression of B7-H3 has been noted in 91.8% of osteosarcoma tissues, which correlated with the increased number of TILs, shorter times of survival, and recurrence in patients [161].

In ChS studies, researchers also identified high expression of CD44 (phagocytic glycoprotein 1, PGP-1)—a tumor stem cell marker, but also a lymphocyte homing receptor and a receptor for hyaluronan, osteopontin (OPN), collagens, and MMPs [33]. CD44 overexpression was typical in high-grade ChSs, and it was correlated with metastatic potential and poor survival [33]. Moreover, CD44 was shown to act as a cofactor for fibroblast growth factor 2 (FGF2), which is related to ChS invasion and its resistance to doxorubicin [33,162].

Similarly to many other tumors, including sarcomas or melanoma, New York esophageal squamous cell carcinoma 1 (NY-ESO-1), a well-known cancer-testis antigen (CTA), has also been found in ChS [163]. Its level was increased in conventional and dedifferentiated ChS, in contrast to clear cell and mesenchymal ChS [164]. NY-ESO-1 can cause spontaneous humoral and cellular immune responses, which, together with its restricted expression pattern, have made it another good potential candidate for cancer immunotherapy [163]. This fact has also been reported in the treatment of ChS cells with 5-aza-2-deoxycitabine, which increased the expression of some CTAs, and yielded NY-ESO-1 and preferentially expressed antigen of melanoma (PRAME) specific CD8+ T cells to recognize and lyse ChS cell lines [165].

The best effects in ChS treatment, using immunotherapy, were observed in combined therapies in dedifferentiated ChS [10,150]. Despite some preclinical trials of multiple immune checkpoint blockades, there were no satisfying effects and not enough data. For this reason, there is still an urgent need to find new immune checkpoints as a future goal in directed ChS treatment.

## 8. Conclusions

ChS is a very rare and still not well-characterized sarcoma subtype. It is highly heterogeneous and is difficult to treat. Although much has already been discovered in many areas of tumor genetics, there are still many unknowns. The biology of ChS is challenging to investigate because of its low frequency of occurrence. For these reasons, there is still a strong need for further research on the molecular bases of ChS. This could be helpful in searching for new therapies and prognostic factors and may help to improve the treatment of these tumors in the future.

## Figures and Tables

**Table 1:** Genetic fusions and chromosomal aberrations in chondrosarcoma. Modified and based on [66].

Sarcoma Type	Genes	Chromosomal Aberrations
Soft tissue chondroma	*HMGA2-LPP* ^a^	t (3;12) (q28; 214)
Mesenchymal chondrosarcoma	*HEY1-NCOA2* ^b^	del (8) (q13; q21)
	*IRFBP2-CDX1* ^c^	t (1;5) (q42; q32)

^a^ High mobility group A2 (*HMGA2*); LIM domain containing preferred translocation partner in lipoma/lipoma preferred partner (*LPP*). ^b^ Hairy/enhancer-of-split related with YRPW motif protein 1 (*HEY-1*); nuclear receptor coactivator 2 (*NCOA2*). ^c^ Interferon regulatory factor 2-binding protein 2 (*IRFBP2*); caudal type homeobox 1 (*CDX1*).

## Data Availability

Not applicable.

## Abbreviations

Antigen-presenting cell (APC), B7 homolog 3 (B7-H3), chondrosarcoma (ChS), collagen type II alpha 1 chain (COL2A1), colony stimulating factor 1 receptor (CSF1R), cytotoxic T-lymphocyte-associated protein 4 (CTLA-4), fibroblast growth factor 2 (FGF2), fibroblast growth factor receptor (FGFR), forkhead box P3 (Foxp3), glioma-associated oncogene (GLI), human leukocyte antigen 1 (HLA-1), Indian Hedgehog Homolog (IHH), isocitrate dehydrogenase (*IDH*), lymphocyte activation gene-3 (LAG-3), mouse double minute 2 homolog (MDM2), major histocompatibility complex (MHC), matrix metalloproteinase (MMP), New York esophageal squamous cell carcinoma 1 (NY-ESO-1), osteopontin (OPN), programmed cell death receptor-1 (PD-1), programmed cell death receptor-1/2 ligand (PDL-1/PDL-2), protein patched homolog 1 (*PTCH1*), signal-regulatory protein alpha (SIRPA), Smoothened, Frizzled Class Receptor (SMO), Sex Determining Region Y-Box (SOX), T cell receptor (TCR), T cell immunoglobulin and mucin domain-containing protein 3 (TIM-3), regulatory T cell (TREG), tuberous sclerosis 1 (*TSC1*), and vascular endothelial growth factor (VEGF).

## References

[1] Leddy L.R., Holmes R.E. (2014). Chondrosarcoma of Bone. Orthopaedic Oncology.

[2] Murphey M.D., Walker E.A., Wilson A.J., Kransdorf M.J., Temple H.T., Gannon F.H. (2003). From the Archives of the AFIP. RadioGraphics.

[3] Fletcher C.D.M., Unni K.K., Mertens F. (2002). World Health Organization Classification of Tumors: Pathology and Genetics of Tumors of Soft Tissue and Bone.

[4] Limaiem F., Davis D.D., Sticco K.L. (2020). Cancer, Chondrosarcoma. https://www.ncbi.nlm.nih.gov/books/NBK538132/.

[5] Heck R.K., Peabody T.D., Simon M.A. (2006). Staging of Primary Malignancies of Bone. CA Cancer J. Clin..

[6] Kim M.-J., Cho K.-J., Ayala A.G., Ro J.Y. (2011). Chondrosarcoma: With updates on molecular genetics. Sarcoma.

[7] Lokuhetty D., White V.A., Cree I.A. (2020). Soft Tissue and Bone Tumours WHO Classification of Tumours.

[8] Evans H.L., Ayala A.G., Romsdahl M.M. (1977). Prognostic factors in chondrosarcoma of bone.A clinicopathologic analysis with emphasis on histologic grading. Cancer.

[9] Grimer R.J., Gosheger G., Taminiau A., Biau D., Matejovsky Z., Kollender Y., San-Julian M., Gherlinzoni F., Ferrari C. (2007). Dedifferentiated chondrosarcoma: Prognostic factors and outcome from a European group. Eur. J. Cancer.

[10] Chow W.A. (2018). Chondrosarcoma: Biology, genetics, and epigenetics. F1000Research.

[11] Shakked R.J., Geller D.S., Gorlick R., Dorfman H.D. (2012). Mesenchymal Chondrosarcoma: Clinicopathologic Study of 20 Cases. Arch. Pathol. Lab. Med..

[12] Italiano A., Mir O., Cioffi A., Palmerini E., Piperno-Neumann S., Perrin C., Chaigneau L., Penel N., Duffaud F., Kurtz J.E. (2013). Advanced chondrosarcomas: Role of chemotherapy and survival. Ann. Oncol..

[13] Mitchell A.D., Ayoub K., Mangham D.C., Grimer R.J., Carter S.R., Tillman R.M. (2000). Experience in the treatment of dedifferentiated chondrosarcoma. J. Bone Jt. Surg..

[14] Rutkowski P., Mazurkiewicz T., Kotrych D., Krzakowski M., Klepacka T., Ługowska I., Grzesiakowska U., Falkowski S., Świtaj T., Szacht M.M. (2016). Zalecenia postępowania diagnostyczno-terapeutycznego u chorych na pierwotne nowotwory złośliwe kości Recommendations for diagnostics and therapy of patients with primary malignant bone tumors. Chir. Narz. Ruchu Ortop. Pol..

[15] Tawbi H.A., Burgess M., Bolejack V., Van Tine B.A., Schuetze S.M., Hu J., D’Angelo S., Attia S., Riedel R.F., Priebat D.A. (2017). Pembrolizumab in advanced soft-tissue sarcoma and bone sarcoma (SARC028): A multicentre, two-cohort, single-arm, open-label, phase 2 trial. Lancet Oncol..

[16] Pan Y., Ma S., Cao K., Zhou S., Zhao A., Li M., Qian F., Zhu C. (2018). Therapeutic approaches targeting cancer stem cells. J. Cancer Res. Ther..

[17] Kuşoğlu A., Biray Avcı Ç. (2019). Cancer stem cells: A brief review of the current status. Gene.

[18] Barbato L., Bocchetti M., Di Biase A., Regad T. (2019). Cancer Stem Cells and Targeting Strategies. Cells.

[19] Erkisa M., Karakas D., Ulukayad E. (2019). Cancer stem cells: Root of the evil. Crit. Rev. Oncog..

[20] Ayob A.Z., Ramasamy T.S. (2018). Cancer stem cells as key drivers of tumour progression. J. Biomed. Sci..

[21] Martínez-Delgado P., Lacerenza S., Obrador-Hevia A., Lopez-Alvarez M., Mondaza-Hernandez J.L., Blanco-Alcaina E., Sanchez-Bustos P., Hindi N., Moura D.S., Martin-Broto J. (2020). Cancer Stem Cells in Soft-Tissue Sarcomas. Cells.

[22] Genadry K.C., Pietrobono S., Rota R., Linardic C.M. (2018). Soft Tissue Sarcoma Cancer Stem Cells: An Overview. Front. Oncol..

[23] Trucco M., Loeb D. (2012). Sarcoma Stem Cells: Do We Know What We Are Looking for?. Sarcoma.

[24] Najafi M., Farhood B., Mortezaee K. (2019). Cancer stem cells (CSCs) in cancer progression and therapy. J. Cell. Physiol..

[25] Was H., Krol S.K., Rotili D., Mai A., Wojtas B., Kaminska B., Maleszewska M. (2019). Histone deacetylase inhibitors exert anti-tumor effects on human adherent and stem-like glioma cells 11 Medical and Health Sciences 1112 Oncology and Carcinogenesis. Clin. Epigenet..

[26] Król S.K., Kaczmarczyk A., Wojnicki K., Wojtas B., Gielniewski B., Grajkowska W., Kotulska K., Szczylik C., Czepko R., Banach M. (2020). Aberrantly Expressed RECQL4 Helicase Supports Proliferation and Drug Resistance of Human Glioma Cells and Glioma Stem Cells. Cancers.

[27] Shibata M., Hoque M.O. (2019). Targeting Cancer Stem Cells: A Strategy for Effective Eradication of Cancer. Cancers.

[28] Yang L., Shi P., Zhao G., Xu J., Peng W., Zhang J., Zhang G., Wang X., Dong Z., Chen F. (2020). Targeting Cancer Stem Cell Pathways for Cancer Therapy.

[29] David E., Blanchard F., Heymann M.F., De Pinieux G., Gouin F., Rédini F., Heymann D. (2011). The Bone Niche of Chondrosarcoma: A Sanctuary for Drug Resistance, Tumour Growth and also a Source of New Therapeutic Targets. Sarcoma.

[30] Diaz-Romero J., Romeo S., Bovée J.V.M.G., Hogendoorn P.C.W., Heini P.F., Mainil-Varlet P. (2010). Hierarchical clustering of flow cytometry data for the study of conventional central chondrosarcoma. J. Cell. Physiol..

[31] Dominici M., Le Blanc K., Mueller I., Slaper-Cortenbach I., Marini F.C., Krause D.S., Deans R.J., Keating A., Prockop D.J., Horwitz E.M. (2006). Minimal criteria for defining multipotent mesenchymal stromal cells. The International Society for Cellular Therapy position statement. Cytotherapy.

[32] Fujiwara T., Ozaki T. (2016). Overcoming Therapeutic Resistance of Bone Sarcomas: Overview of the Molecular Mechanisms and Therapeutic Targets for Bone Sarcoma Stem Cells. Stem Cells Int..

[33] Boehme K., Schleicher S., Traub F., Rolauffs B. (2018). Chondrosarcoma: A Rare Misfortune in Aging Human Cartilage? The Role of Stem and Progenitor Cells in Proliferation, Malignant Degeneration and Therapeutic Resistance. Int. J. Mol. Sci..

[34] Alexander D., Schäfer F., Munz A., Friedrich B., Klein C., Hoffmann J., Bühring H.J., Reinert S. (2009). LNGFR induction during Osteogenesis of human jaw periosteum-derived cells. Cell. Physiol. Biochem..

[35] Wirths S., Malenke E., Kluba T., Rieger S., Müller M.R., Schleicher S., von Weyhern C.H., Nagl F., Fend F., Vogel W. (2013). Shared Cell Surface Marker Expression in Mesenchymal Stem Cells and Adult Sarcomas. Stem Cells Transl. Med..

[36] Tian J., Li X., Si M., Liu T., Li J. (2014). CD271+ Osteosarcoma Cells Display Stem-Like Properties. PLoS ONE.

[37] Andrzejewska A., Lukomska B., Janowski M. (2019). Concise Review: Mesenchymal Stem Cells: From Roots to Boost. Stem Cells.

[38] Gibbs C.P., Kukekov V.G., Reith J.D., Tchigrinova O., Suslov O.N., Scott E.W., Ghivizzani S.C., Ignatova T.N., Steindler D.A. (2005). Stem-like cells in bone sarcomas: Implications for tumorigenesis. Neoplasia.

[39] Senbanjo L.T., Chellaiah M.A. (2017). CD44: A Multifunctional Cell Surface Adhesion Receptor Is a Regulator of Progression and Metastasis of Cancer Cells. Front. Cell Dev. Biol..

[40] Heyse T.J., Malcherczyk D., Moll R., Timmesfeld N., Wapelhorst J., Fuchs-Winkelmann S., Paletta J.R.J., Schofer M.D. (2010). CD44: Survival and metastasis in chondrosarcoma. Osteoarthr. Cartil..

[41] Tirino V., Desiderio V., D’Aquino R., De Francesco F., Pirozzi G., Galderisi U., Cavaliere C., De Rosa A., Papaccio G. (2008). Detection and characterization of CD133+ cancer stem cells in human solid tumours. PLoS ONE.

[42] Tirino V., Desiderio V., Paino F., De Rosa A., Papaccio F., Fazioli F., Pirozzi G., Papaccio G. (2011). Human primary bone sarcomas contain CD133 + cancer stem cells displaying high tumorigenicity in vivo. FASEB J..

[43] Clark D.W., Palle K. (2016). Aldehyde dehydrogenases in cancer stem cells: Potential as therapeutic targets. Ann. Transl. Med..

[44] Menendez S.T., Rey V., Martinez-Cruzado L., Gonzalez M.V., Morales-Molina A., Santos L., Blanco V., Alvarez C., Estupiñan O., Allonca E. (2020). SOX2 Expression and Transcriptional Activity Identifies a Subpopulation of Cancer Stem Cells in Sarcoma with Prognostic Implications. Cancers.

[45] Silva J., Nichols J., Theunissen T.W., Guo G., van Oosten A.L., Barrandon O., Wray J., Yamanaka S., Chambers I., Smith A. (2009). Nanog Is the Gateway to the Pluripotent Ground State. Cell.

[46] Baek K.H., Choi J., Pei C.Z. (2020). Cellular functions of OCT-3/4 regulated by ubiquitination in proliferating cells. Cancers.

[47] Li J., Shen J., Wang K., Hornicek F., Duan Z. (2016). The Roles of Sox Family Genes in Sarcoma. Curr. Drug Targets.

[48] Liu P., Shen J.K., Xu J., Trahan C.A., Hornicek F.J., Duan Z. (2016). Aberrant DNA methylations in chondrosarcoma. Epigenomics.

[49] Armstrong L., Stojkovic M., Dimmick I., Ahmad S., Stojkovic P., Hole N., Lako M. (2004). Phenotypic Characterization of Murine Primitive Hematopoietic Progenitor Cells Isolated on Basis of Aldehyde Dehydrogenase Activity. Stem Cells.

[50] Vassalli G. (2019). Aldehyde dehydrogenases: Not just markers, but functional regulators of stem cells. Stem Cells Int..

[51] Sládek N.E. (2003). Human aldehyde dehydrogenases: Potential pathological, pharmacological, and toxicological impact. J. Biochem. Mol. Toxicol..

[52] Rey V., Menendez S.T., Estupiñan O., Rodriguez A., Santos L., Tornin J., Martinez-Cruzado L., Castillo D., Ordoñez G.R., Costilla S. (2019). New Chondrosarcoma Cell Lines with Preserved Stem Cell Properties to Study the Genomic Drift During In Vitro/In Vivo Growth. J. Clin. Med..

[53] Hoyt A.K., Moran A., Granger C., Sedani A., Saigh S., Brown J., Galoian K.A. (2019). PRP-1 significantly decreases the ALDHhigh cancer stem cell population and regulates the aberrant Wnt/β-catenin pathway in human chondrosarcoma JJ012 cells. Oncol. Rep..

[54] Vares G., Ahire V., Sunada S., Ho Kim E., Sai S., Chevalier F., Romeo P.H., Yamamoto T., Nakajima T., Saintigny Y. (2020). A multimodal treatment of carbon ions irradiation, miRNA-34 and mTOR inhibitor specifically control high-grade chondrosarcoma cancer stem cells. Radiother. Oncol..

[55] Misso G., Di Martino M.T., De Rosa G., Farooqi A.A., Lombardi A., Campani V., Zarone M.R., Gullà A., Tagliaferri P., Tassone P. (2014). Mir-34: A new weapon against cancer?. Mol. Ther. Nucleic Acids.

[56] Guessous F., Zhang Y., Kofman A., Catania A., Li Y., Schiff D., Purow B., Abounader R. (2010). microRNA-34a is tumor suppressive in brain tumors and glioma stem cells. Cell Cycle.

[57] Liu C., Kelnar K., Liu B., Chen X., Calhoun-Davis T., Li H., Patrawala L., Yan H., Jeter C., Honorio S. (2011). Identification of miR-34a as a potent inhibitor of prostate cancer progenitor cells and metastasis by directly repressing CD44 HHS Public Access. Nat. Med..

[58] Nalls D., Tang S.-N., Rodova M., Srivastava R.K., Shankar S. (2011). Targeting Epigenetic Regulation of miR-34a for Treatment of Pancreatic Cancer by Inhibition of Pancreatic Cancer Stem Cells. PLoS ONE.

[59] Kim H., Cho Y., Kim H.-S., Kang D., Cheon D., Kim Y.-J., Chang M.J., Lee K.M., Chang C.B., Kang S.-B. (2020). A system-level approach identifies HIF-2α as a critical regulator of chondrosarcoma progression. Nat. Commun..

[60] Chen C., Ma Q., Ma X., Liu Z., Liu X. (2011). Association of Elevated HIF-2α Levels with Low Beclin 1 Expression and Poor Prognosis in Patients with Chondrosarcoma. Ann. Surg. Oncol..

[61] Chen C., Zhou H., Wei F., Jiang L., Liu X., Liu Z., Ma Q. (2011). Increased levels of hypoxia-inducible factor-1α are associated with Bcl-xL expression, tumor apoptosis, and clinical outcome in chondrosarcoma. J. Orthop. Res..

[62] Oshiro Y., Chaturvedi V., Hayden D., Nazeer T., Johnson M., Johnston D.A., Ordóñez N.G., Ayala A.G., Czerniak B. (1998). Altered p53 is associated with aggressive behavior of chondrosarcoma. Cancer.

[63] Yamaguchi T., Toguchida J., Wadayama B., Kanoe H., Nakayama T., Ishizaki K., Ikenaga M., Kotoura Y., Sasaki M.S. (1996). Loss of heterozygosity and tumor suppressor gene mutations in chondrosarcomas. Anticancer Res..

[64] Terek R.M., Healey J.H., Garin-Chesa P., Mak S., Huvos A., Albino A.P. (1998). p53 mutations in chondrosarcoma. Diagn. Mol. Pathol..

[65] van Beerendonk H.M., Rozeman L.B., Taminiau A.H., Sciot R., Bovée J.V., Cleton-Jansen A.-M., Hogendoorn P.C. (2004). Molecular analysis of the INK4A/INK4A-ARF gene locus in conventional(central) chondrosarcomas and enchondromas: Indication of an important gene for tumour progression. J. Pathol..

[66] Fiedorowicz M., Bartnik E., Sobczuk P., Teterycz P., Czarnecka A.M. (2018). Molecular biology of sarcoma. Oncol. Clin. Pract..

[67] Hallor K.H., Staaf J., Bovee J.V.M.G., Hogendoorn P.C.W., Cleton-Jansen A.-M., Knuutila S., Savola S., Niini T., Brosjo O., Bauer H.C.F. (2009). Genomic Profiling of Chondrosarcoma: Chromosomal Patterns in Central and Peripheral Tumors. Clin. Cancer Res..

[68] Sandberg A.A., Bridge J.A. (2003). Updates on the cytogenetics and molecular genetics of bone and soft tissue tumors. Cancer Genet. Cytogenet..

[69] Miettinen M., Felisiak-Golabek A., Luiña Contreras A., Glod J., Kaplan R.N., Killian J.K., Lasota J. (2019). New fusion sarcomas: Histopathology and clinical significance of selected entities. Hum. Pathol..

[70] Sandberg A.A. (2004). Genetics of chondrosarcoma and related tumors. Curr. Opin. Oncol..

[71] Agaram N.P., Zhang L., Sung Y.-S., Singer S., Antonescu C.R. (2014). Extraskeletal myxoid chondrosarcoma with non–EWSR1-NR4A3 variant fusions correlate with rhabdoid phenotype and high-grade morphology. Hum. Pathol..

[72] Broehm C.J., Wu J., Gullapalli R.R., Bocklage T. (2014). Extraskeletal myxoid chondrosarcoma with a t(9;16)(q22;p11.2) resulting in a NR4A3-FUS fusion. Cancer Genet..

[73] Paioli A., Stacchiotti S., Campanacci D., Palmerini E., Frezza A.M., Longhi A., Radaelli S., Donati D.M., Beltrami G., Bianchi G. (2020). Extraskeletal Myxoid Chondrosarcoma with Molecularly Confirmed Diagnosis: A Multicenter Retrospective Study Within the Italian Sarcoma Group. Ann. Surg. Oncol..

[74] Stacchiotti S., Baldi G.G., Morosi C., Gronchi A., Maestro R. (2020). Extraskeletal Myxoid Chondrosarcoma: State of the Art and Current Research on Biology and Clinical Management. Cancers.

[75] Sbaraglia M., Dei Tos A.P. (2019). The pathology of soft tissue sarcomas. Radiol. Med..

[76] Wang L., Motoi T., Khanin R., Olshen A., Mertens F., Bridge J., Cin P.D., Antonescu C.R., Singer S., Hameed M. (2012). Identification of a novel, recurrent HEY1-NCOA2 fusion in mesenchymal chondrosarcoma based on a genome-wide screen of exon-level expression data. Genes Chromosomes Cancer.

[77] Li L., Hu X., Eid J.E., Rosenberg A.E., Wilky B.A., Ban Y., Sun X., Galoian K., DeSalvo J., Yue J. (2020). Mutant IDH1 Depletion Downregulates Integrins and Impairs Chondrosarcoma Growth. Cancers.

[78] Lugowska I., Teterycz P., Mikula M., Kulecka M., Kluska A., Balabas A., Piatkowska M., Wagrodzki M., Pienkowski A., Rutkowski P. (2018). IDH1/2 Mutations Predict Shorter Survival in Chondrosarcoma. J. Cancer.

[79] Dang L., White D.W., Gross S., Bennett B.D., Bittinger M.A., Driggers E.M., Fantin V.R., Jang H.G., Jin S., Keenan M.C. (2009). Cancer-associated IDH1 mutations produce 2-hydroxyglutarate. Nature.

[80] Venneker S., Kruisselbrink A.B., Baranski Z., Palubeckaite I., Briaire-de Bruijn I.H., Oosting J., French P.J., Danen E.H.J., Bovée J.V.M.G. (2020). Beyond the Influence of IDH Mutations: Exploring Epigenetic Vulnerabilities in Chondrosarcoma. Cancers.

[81] Zhang H., Wei Q., Tsushima H., Puviindran V., Tang Y.J., Pathmanapan S., Poon R., Ramu E., Al-Jazrawe M., Wunder J. (2019). Intracellular cholesterol biosynthesis in enchondroma and chondrosarcoma. JCI Insight.

[82] Amary M.F., Bacsi K., Maggiani F., Damato S., Halai D., Berisha F., Pollock R., O’Donnell P., Grigoriadis A., Diss T. (2011). IDH1 and IDH2 mutations are frequent events in central chondrosarcoma and central and periosteal chondromas but not in other mesenchymal tumours. J. Pathol..

[83] Tarpey P.S., Behjati S., Cooke S.L., Van Loo P., Wedge D.C., Pillay N., Marshall J., O’Meara S., Davies H., Nik-Zainal S. (2013). Frequent mutation of the major cartilage collagen gene COL2A1 in chondrosarcoma. Nat. Genet..

[84] Nicolle R., Ayadi M., Gomez-Brouchet A., Armenoult L., Banneau G., Elarouci N., Tallegas M., Decouvelaere A.V., Aubert S., Rédini F. (2019). Integrated molecular characterization of chondrosarcoma reveals critical determinants of disease progression. Nat. Commun..

[85] Cote G.M., He J., Choy E. (2018). Next-Generation Sequencing for Patients with Sarcoma: A Single Center Experience. Oncologist.

[86] Olivier M., Hollstein M., Hainaut P. (2010). TP53 Mutations in Human Cancers: Origins, Consequences, and Clinical Use. Cold Spring Harb. Perspect. Biol..

[87] Baugh E.H., Ke H., Levine A.J., Bonneau R.A., Chan C.S. (2018). Why are there hotspot mutations in the TP53 gene in human cancers?. Cell Death Differ..

[88] Nazeri E., Gouran Savadkoohi M., Majidzadeh-A K., Esmaeili R. (2018). Chondrosarcoma: An overview of clinical behavior, molecular mechanisms mediated drug resistance and potential therapeutic targets. Crit. Rev. Oncol. Hematol..

[89] Lam S.W., Langevelde K., Suurmeijer A.J.H., Cleven A.H.G., Bovée J.V.M.G. (2019). Conventional chondrosarcoma with focal clear cell change: A clinicopathological and molecular analysis. Histopathology.

[90] Bovée J.V.M.G., Hogendoorn P.C.W., Wunder J.S., Alman B.A. (2010). Cartilage tumours and bone development: Molecular pathology and possible therapeutic targets. Nat. Rev. Cancer.

[91] Kaminska B., Czapski B., Guzik R., Król S.K., Gielniewski B. (2019). Consequences of IDH1/2 mutations in gliomas and an assessment of inhibitors targeting mutated IDH proteins. Molecules.

[92] Cleven A.H.G., Suijker J., Agrogiannis G., Briaire-de Bruijn I.H., Frizzell N., Hoekstra A.S., Wijers-Koster P.M., Cleton-Jansen A.-M., Bovée J.V.M.G. (2017). IDH1 or -2 mutations do not predict outcome and do not cause loss of 5-hydroxymethylcytosine or altered histone modifications in central chondrosarcomas. Clin. Sarcoma Res..

[93] Suijker J., Oosting J., Koornneef A., Struys E.A., Salomons G.S., Schaap F.G., Waaijer C.J.F., Wijers-Koster P.M., Briaire-de Bruijn I.H., Haazen L. (2015). Inhibition of mutant IDH1 decreases D-2-HG levels without affecting tumorigenic properties of chondrosarcoma cell lines. Oncotarget.

[94] Hamm C.A., Stevens J.W., Xie H., Vanin E.F., Morcuende J.A., Abdulkawy H., Seftor E.A., Sredni S.T., Bischof J.M., Wang D. (2010). Microenvironment alters epigenetic and gene expression profiles in Swarm rat chondrosarcoma tumors. BMC Cancer.

[95] Asp J., Angiorgi L.S., Nerot S.E.I., Indahl A.L., Olendini L.M., Enassi M.S.B., Icci P.P. (2000). Changes of the p16 gene but not the p53 gene in human chondrosarcoma tissues. Int. J. Cancer.

[96] Röpke M., Boltze C., Neumann H.W., Roessner A., Schneider-Stock R. (2003). Genetic and epigenetic alterations in tumor progression in a dedifferentiated chondrosarcoma. Pathol. Res. Pract..

[97] Jin Z., Han Y.X., Han X.R. (2013). Loss of RUNX3 expression may contribute to poor prognosis in patients with chondrosarcoma. J. Mol. Histol..

[98] Bui C., Ouzzine M., Talhaoui I., Sharp S., Prydz K., Coughtrie M.W.H., Fournel-Gigleux S. (2010). Epigenetics: Methylation-associated repression of heparan sulfate 3- O -sulfotransferase gene expression contributes to the invasive phenotype of H-EMC-SS chondrosarcoma cells. FASEB J..

[99] Peterse E.F.P., Van Den Akker B.E.W.M., Niessen B., Oosting J., Suijker J., De Jong Y., Danen E.H.J., Cleton-Jansen A.M., Bovée J.V.M.G. (2017). NAD Synthesis Pathway Interference Is a Viable Therapeutic Strategy for Chondrosarcoma. Mol. Cancer Res..

[100] Bovolenta P., Esteve P., Ruiz J.M., Cisneros E., Lopez-Rios J. (2008). Beyond Wnt inhibition: New functions of secreted Frizzled-related proteins in development and disease. J. Cell Sci..

[101] Sheng W., Zhang Z.C., Shi D.Y., Wang B.C., Wu Q., Shao Z.W., Yang S.H., He T.C., Liu J.X. (2018). Epigenetic silencing of SFRP5 promotes the metastasis and invasion of chondrosarcoma by expression inhibition and Wnt signaling pathway activation. Chem. Biol. Interact..

[102] Hamm C.A., Xie H., Costa F.F., Vanin E.F., Seftor E.A., Sredni S.T., Bischof J., Wang D., Bonaldo M.F., Hendrix M.J.C. (2009). Global demethylation of rat chondrosarcoma cells after treatment with 5-Aza-2′-deoxycytidine results in increased tumorigenicity. PLoS ONE.

[103] Domann F.E., Fitzgerald M.P., Gourronc F., Teoh M.L.T., Provenzano M.J., Case A.J., Martin J.A. (2011). Human chondrosarcoma cells acquire an epithelial-like gene expression pattern via an epigenetic switch: Evidence for mesenchymal-epithelial transition during sarcomagenesis. Sarcoma.

[104] Jancalek R. (2014). The role of the TP73 gene and its transcripts in neuro-oncology. Br. J. Neurosurg..

[105] Liu P., Garbutt C., Hornicek F.J., Liu F., Duan Z. (2017). Aberration of p73 promoter methylation in chondrosarcoma. Anticancer Res..

[106] Hosseini A., Minucci S. (2018). Alterations of Histone Modifications in Cancer. Epigenetics in Human Disease.

[107] Shanmugam M.K., Arfuso F., Arumugam S., Chinnathambi A., Jinsong B., Warrier S., Wang L.Z., Kumar A.P., Ahn K.S., Sethi G. (2018). Role of novel histone modifications in cancer. Oncotarget.

[108] Zhao Z., Shilatifard A. (2019). Epigenetic modifications of histones in cancer. Genome Biol..

[109] Ververis K., Hiong A., Karagiannis T.C., Licciardi P. (2013). V Histone deacetylase inhibitors (HDACIs): Multitargeted anticancer agents. Biologics.

[110] Mottamal M., Zheng S., Huang T., Wang G. (2015). Histone Deacetylase Inhibitors in Clinical Studies as Templates for New Anticancer Agents. Molecules.

[111] Feng H., Wang J., Xu J., Xie C., Gao F., Li Z. (2017). The expression of SIRT1 regulates the metastaticplasticity of chondrosarcoma cells by inducing epithelial-mesenchymal transition. Sci. Rep..

[112] Zhu J., Gu J., Ma J., Xu Z., Tao H. (2015). Histone deacetylase inhibitors repress chondrosarcoma cell proliferation. J. Buon..

[113] Yamamoto S., Tanaka K., Sakimura R., Okada T., Nakamura T., Li Y., Takasaki M., Nakabeppu Y., Iwamoto Y. (2008). Suberoylanilide hydroxamic acid (SAHA) induces apoptosis or autophagy-associated cell death in chondrosarcoma cell lines. Anticancer Res..

[114] Sakimura R., Tanaka K., Yamamoto S., Matsunobu T., Li X., Hanada M., Okada T., Nakamura T., Li Y., Iwamoto Y. (2007). The Effects of Histone Deacetylase Inhibitors on the Induction of Differentiation in Chondrosarcoma Cells. Clin. Cancer Res..

[115] Röpke M., Boltze C., Meyer B., Neumann H.W., Roessner A., Schneider-Stock R. (2006). Rb-loss is associated with high malignancy in chondrosarcoma. Oncol. Rep..

[116] Schrage Y.M., Lam S., Jochemsen A.G., Cleton-Jansen A.-M., Taminiau A.H.M., Hogendoorn P.C.W., Bovée J.V.M.G. (2009). Central chondrosarcoma progression is associated with pRb pathway alterations: CDK4 down-regulation and p16 overexpression inhibit cell growth in vitro. J. Cell. Mol. Med..

[117] Ho L., Stojanovski A., Whetstone H., Wei Q.X., Mau E., Wunder J.S., Alman B. (2009). Gli2 and p53 Cooperate to Regulate IGFBP-3- Mediated Chondrocyte Apoptosis in the Progression from Benign to Malignant Cartilage Tumors. Cancer Cell.

[118] Ray-Coquard I., Blay J.Y., Italiano A., Le Cesne A., Penel N., Zhi J., Heil F., Rueger R., Graves B., Ding M. (2012). Effect of the MDM2 antagonist RG7112 on the P53 pathway in patients with MDM2-amplified, well-differentiated or dedifferentiated liposarcoma: An exploratory proof-of-mechanism study. Lancet Oncol..

[119] Sherr C.J., McCormick F. (2002). The RB and p53 pathways in cancer. Cancer Cell.

[120] Ianari A., Natale T., Calo E., Ferretti E., Alesse E., Screpanti I., Haigis K., Gulino A., Lees J.A. (2009). Proapoptotic Function of the Retinoblastoma Tumor Suppressor Protein. Cancer Cell.

[121] Roberts P.J., Kumarasamy V., Witkiewicz A.K., Knudsen E.S. (2020). Chemotherapy and CDK4/6 Inhibitors: Unexpected Bedfellows. Mol. Cancer Ther..

[122] Engelman J.A. (2009). Targeting PI3K signalling in cancer: Opportunities, challenges and limitations. Nat. Rev. Cancer.

[123] Schrage Y.M., Briaire-de Bruijn I.H., De Miranda N.F.C.C., Van Oosterwijk J., Taminiau A.H.M., Van Wezel T., Hogendoorn P.C.W., Bovée J.V.M.G. (2009). Kinome profiling of chondrosarcoma reveals Src-pathway activity and dasatinib as option for treatment. Cancer Res..

[124] Jang J.H., Chung C.P. (2005). Tenascin-C promotes cell survival by activation of Akt in human chondrosarcoma cell. Cancer Lett..

[125] Lin C., Meitner P.A., Terek R.M. (2002). PTEN mutation is rare in chondrosarcoma. Diagnostic Mol. Pathol..

[126] LoRusso P.M. (2016). Inhibition of the PI3K/AKT/mTOR pathway in solid tumors. J. Clin. Oncol..

[127] Van Oosterwijk J.G., Van Ruler M.A.J.H., Briaire-De Bruijn I.H., Herpers B., Gelderblom H., Van De Water B., Bovée J.V.M.G. (2013). Src kinases in chondrosarcoma chemoresistance and migration: Dasatinib sensitises to doxorubicin in TP53 mutant cells. Br. J. Cancer.

[128] Schuetze S., Wathen J.K., Lucas D.R., Choy E., Samuels B.L. (2016). SARC009: Phase 2 study of dasatinib in patients with previously treated, high-grade, advanced sarcoma. Cancer.

[129] Gibbons J.J., Abraham R.T., Yu K. (2009). Mammalian Target of Rapamycin: Discovery of Rapamycin Reveals a Signaling Pathway Important for Normal and Cancer Cell Growth. Semin. Oncol..

[130] Sun S.Y.E. (2013). mTOR kinase inhibitors as potential cancer therapeutic drugs. Cancer Lett..

[131] Zhang Y.X., Van Oosterwijk J.G., Sicinska E., Moss S., Remillard S.P., Van Wezel T., Bühnemann C., Hassan A.B., Demetri G.D., Bovée J.V.M.G. (2013). Functional profiling of receptor tyrosine kinases and downstream signaling in human chondrosarcomas identifies pathways for rational targeted therapy. Clin. Cancer Res..

[132] Galoian K., Temple T.H., Galoyan A. (2011). Cytostatic effect of the hypothalamic cytokine prp-1 is mediated by mTOR and cMYc inhibition in high grade chondrosarcoma. Neurochem. Res..

[133] Wu M.H., Lo J.F., Kuo C.H., Lin J.A., Lin Y.M., Chen L.M., Tsai F.J., Tsai C.H., Huang C.Y., Tang C.H. (2012). Endothelin-1 promotes MMP-13 production and migration in human chondrosarcoma cells through FAK/PI3K/Akt/mTOR pathways. J. Cell. Physiol..

[134] Perez J., Decouvelaere A.V., Pointecouteau T., Pissaloux D., Michot J.P., Besse A., Blay J.Y., Dutour A. (2012). Inhibition of chondrosarcoma growth by mTOR inhibitor in an in vivo syngeneic rat model. PLoS ONE.

[135] Merimsky O., Bernstein-Molho R., Sagi-Eisenberg R. (2008). Targeting the mammalian target of rapamycin in myxoid chondrosarcoma. Anticancer. Drugs.

[136] Bernstein-Molho R., Kollender Y., Issakov J., Bickels J., Dadia S., Flusser G., Meller I., Sagi-Eisenberg R., Merimsky O. (2012). Clinical activity of mTOR inhibition in combination with cyclophosphamide in the treatment of recurrent unresectable chondrosarcomas. Cancer Chemother. Pharmacol..

[137] Cannonier S., Sterling J. (2015). The Role of Hedgehog Signaling in Tumor Induced Bone Disease. Cancers.

[138] Sakamoto A. (2014). The molecular pathogenesis of dedifferentiated chondrosarcoma. Indian J. Orthop..

[139] Campbell V.T., Nadesan P., Ali S.A., Wang C.Y.Y., Whetstone H., Poon R., Wei Q., Keilty J., Proctor J., Wang L.W. (2014). Hedgehog Pathway Inhibition in Chondrosarcoma Using the Smoothened Inhibitor IPI-926 Directly Inhibits Sarcoma Cell Growth. Mol. Cancer Ther..

[140] Wagner A.J., Hohenberger P., Okuno S., Eriksson M., Patel S., Ferrari S., Casali P.G., Chawla S.P., Woehr M., Ross R. Results from a Phase 2 Randomized, Placebo-Controlled, Double Blind Study of the Hedgehog Pathway Antagonist IPI-926 in Patients with Advanced Chondrosarcoma. Proceedings of the Connective Tissue Oncology Society 18th Annual Meeting.

[141] Simard F.A., Richert I., Vandermoeten A., Decouvelaere A.-V., Michot J.-P., Caux C., Blay J.-Y., Dutour A. (2017). Description of the immune microenvironment of chondrosarcoma and contribution to progression. Oncoimmunology.

[142] Boxberg M., Steiger K., Lenze U., Rechl H., von Eisenhart-Rothe R., Wörtler K., Weichert W., Langer R., Specht K. (2018). PD-L1 and PD-1 and characterization of tumor-infiltrating lymphocytes in high grade sarcomas of soft tissue—Prognostic implications and rationale for immunotherapy. Oncoimmunology.

[143] Richert I., Gomez-Brouchet A., Bouvier C., Du Bouexic De Pinieux G., Karanian M., Blay J.-Y., Dutour A. (2020). The immune landscape of chondrosarcoma—Potential for therapeutic targeting of CSFR1+ macrophages. J. Bone Oncol..

[144] Aras S., Zaidi M.R. (2017). TAMeless traitors: Macrophages in cancer progression and metastasis. Br. J. Cancer.

[145] Pardoll D.M. (2012). The blockade of immune checkpoints in cancer immunotherapy. Nat. Rev. Cancer.

[146] Simard F. (2016). Implication of Immune System in Chondrosarcoma Progression and Therapeutic Response: Could Immunotherapy Play a Role in Chondrosarcoma Treatment?. Ph.D. Thesis.

[147] Hadrup S., Donia M., Thor Straten P. (2013). Effector CD4 and CD8 T cells and their role in the tumor microenvironment. Cancer Microenviron..

[148] Kostine M., Cleven A.H., de Miranda N.F.C.C., Italiano A., Cleton-Jansen A.-M., Bovée J.V.M.G. (2016). Analysis of PD-L1, T-cell infiltrate and HLA expression in chondrosarcoma indicates potential for response to immunotherapy specifically in the dedifferentiated subtype. Mod. Pathol..

[149] D’Angelo S.P., Shoushtari A.N., Agaram N.P., Kuk D., Qin L.-X., Carvajal R.D., Dickson M.A., Gounder M., Keohan M.L., Schwartz G.K. (2015). Prevalence of tumor-infiltrating lymphocytes and PD-L1 expression in the soft tissue sarcoma microenvironment. Hum. Pathol..

[150] Thanindratarn P., Dean D.C., Nelson S.D., Hornicek F.J., Duan Z. (2019). Advances in immune checkpoint inhibitors for bone sarcoma therapy. J. Bone Oncol..

[151] Zhu Z., Jin Z., Zhang M., Tang Y., Yang G., Yuan X., Yao J., Sun D. (2017). Prognostic value of programmed death-ligand 1 in sarcoma: A meta-analysis. Oncotarget.

[152] Yang X., Zhu G., Yang Z., Zeng K., Liu F., Sun J. (2018). Expression of PD-L1/PD-L2 is associated with high proliferation index of Ki-67 but not with TP53 overexpression in chondrosarcoma. Int. J. Biol. Markers.

[153] Wagner M.J., Ricciotti R.W., Mantilla J., Loggers E.T., Pollack S.M., Cranmer L.D. (2018). Response to PD1 inhibition in conventional chondrosarcoma. J. Immunother. Cancer.

[154] Kim J.R., Moon Y.J., Kwon K.S., Bae J.S., Wagle S., Kim K.M., Park H.S., Lee H., Moon W.S., Chung M.J. (2013). Tumor infiltrating PD1-positive lymphocytes and the expression of PD-L1 predict poor prognosis of soft tissue sarcomas. PLoS ONE.

[155] He Y., Cao J., Zhao C., Li X., Zhou C., Hirsch F. (2018). TIM-3, a promising target for cancer immunotherapy. Onco. Targets. Ther..

[156] Gao X., Zhu Y., Li G., Huang H., Zhang G., Wang F., Sun J., Yang Q., Zhang X., Lu B. (2012). TIM-3 Expression Characterizes Regulatory T Cells in Tumor Tissues and Is Associated with Lung Cancer Progression. PLoS ONE.

[157] Komohara Y., Morita T., Annan D.A., Horlad H., Ohnishi K., Yamada S., Nakayama T., Kitada S., Suzu S., Kinoshita I. (2015). The Coordinated Actions of TIM-3 on Cancer and Myeloid Cells in the Regulation of Tumorigenicity and Clinical Prognosis in Clear Cell Renal Cell Carcinomas. Cancer Immunol. Res..

[158] Andrews L.P., Marciscano A.E., Drake C.G., Vignali D.A.A. (2017). LAG3 (CD223) as a cancer immunotherapy target. Immunol. Rev..

[159] D’Angelo S.P., Tap W.D., Schwartz G.K., Carvajal R.D. (2014). Sarcoma Immunotherapy: Past Approaches and Future Directions. Sarcoma.

[160] Leach D.R., Krummel M.F., Allison J.P. (1996). Enhancement of Antitumor Immunity by CTLA-4 Blockade. Science.

[161] Wang L., Zhang Q., Chen W., Shan B., Ding Y., Zhang G., Cao N., Liu L., Zhang Y. (2013). B7-H3 is Overexpressed in Patients Suffering Osteosarcoma and Associated with Tumor Aggressiveness and Metastasis. PLoS ONE.

[162] Hsieh M., Huang C., Lin C., Tang C., Lin C., Lee I., Huang H., Chen J. (2020). Basic fibroblast growth factor promotes doxorubicin resistance in chondrosarcoma cells by affecting XRCC5 expression. Mol. Carcinog..

[163] Thomas R., Al-Khadairi G., Roelands J., Hendrickx W., Dermime S., Bedognetti D., Decock J. (2018). NY-ESO-1 Based Immunotherapy of Cancer: Current Perspectives. Front. Immunol..

[164] Endo M., de Graaff M.A., Ingram D.R., Lim S., Lev D.C., Briaire-de Bruijn I.H., Somaiah N., Bovée J.V., Lazar A.J., Nielsen T.O. (2015). NY-ESO-1 (CTAG1B) expression in mesenchymal tumors. Mod. Pathol..

[165] Pollack S.M., Li Y., Blaisdell M.J., Farrar E.A., Chou J., Hoch B.L., Loggers E.T., Rodler E., Eary J.F., Conrad E.U. (2012). NYESO-1/LAGE-1s and PRAME Are Targets for Antigen Specific T Cells in Chondrosarcoma following Treatment with 5-Aza-2-Deoxycitabine. PLoS ONE.

